# Addressing Stigma in the Community: A Rights-Based Approach to Community-Building

**DOI:** 10.1093/geroni/igab046.1388

**Published:** 2021-12-17

**Authors:** Deborah O'Connor

**Affiliations:** University of British Columbia, Vancouver, British Columbia, Canada

## Abstract

Article 12 of the United Nations Convention of Rights of Persons with Disabilities (CRPD) affirms the rights of persons with physical and mental disabilities to be treated as equal, and deserving of state support to realize their full human potential. This focus on a ‘positive’ right to support (as opposed to the ‘negative’ right to non-interference) has established an important set of expectations around societal responses to people living with dementia(PLWD). This presentation examines the contributions of a rights-based approach to build community with and for PLWD. Data is drawn from Participatory Action Research (PAR) and bi-weekly online action groups with N=10 PLWD in urban and rural British Columbia. Two thematic targets were identified. First, it is important to bring together PLWD in ways that create a sense of solidarity and inclusion. Second, fostering community requires addressing the stigma and discrimination which often leave PLWD feeling isolated, excluded, and marginalized.

